# Administration of a Decoction of Sucrose- and Polysaccharide-Rich Radix Astragali (*Huang Qi*) Ameliorated Insulin Resistance and Fatty Liver but Affected Beta-Cell Function in Type 2 Diabetic Rats

**DOI:** 10.1155/2011/349807

**Published:** 2011-01-10

**Authors:** Yi-Chen Juan, Yao-Haur Kuo, Chia-Chuan Chang, Li-Jie Zhang, Yan-Yu Lin, Chia-Yun Hsu, Hui-Kang Liu

**Affiliations:** ^1^Institute of Pharmacology, Yang Ming University, Taipei 112, Taiwan; ^2^Division of Herbal Drugs and Natural Products, National Research Institute of Chinese Medicine, Taipei 112, Taiwan; ^3^Division of Medicinal Chemistry, National Research Institute of Chinese Medicine, Taipei 112, Taiwan

## Abstract

The current investigation attempted to confirm the beneficial actions of a chemically characterized Radix Astragali decoction (AM-W) against type 2 diabetic (T2D) Sprague-Dawley (SD) rats. Using a case/control design, after 2 months of treatment with AM-W (500 mg/kg, daily i.p.) in T2D rats therapeutic outcomes were compared. Sucrose and *Astragalus* polysaccharides (ASPs) were shown to exist in nearly equal proportions in AM-W. Body weight loss, an improvement in insulin sensitivity, and an attenuation of fatty liver after AM-W administration in T2D rats were evident. Surprisingly, blood sugar, beta-cell function, and glucose tolerance in T2D rats did not improve with AM-W treatment. Further investigation indicated the deleterious effects of the addition of sucrose (100 and 500 *μ*g/mL) and APSs (500 *μ*g/mL) on glucose-stimulated insulin secretion and viability, respectively. In conclusion, a proper administration dosage and a reduction in the sucrose content are keys to maximizing the merits of this herb.

## 1. Introduction

To manage type 2 diabetes (T2D), both insulin resistance and beta-cell dysfunction are two major pathological factors which need to be controlled [1]. In addition, nonalcoholic fatty liver disease (NAFLD) is strongly associated with yet often overlooked in T2D. Uncontrolled NAFLD might subsequently manifest as hepatic inflammation, steatosis (NASH), cirrhosis, and even carcinogenesis [2].

Traditional Chinese medicine (TCM) frequently prescribes the root of *Astragali membranaceus* (Radix Astragali (RA); *Huang Qi*) in antidiabetes TCM formulae for “qi supplementation” [3]. According to current knowledge, *Astragalus* polysaccharides (APSs) and saponins are two bioactive constituents of RA found to be beneficial for diabetes interventions. Administration of purified APSs can reduce diabetic hyperglycemia and lead to indirect preservation of beta-cell function and mass via immunomodulatory effects on type 1 diabetic mice [4, 5]. Recent studies also showed that purified APSs partially restore glucose homeostasis by an insulin-sensitizing effect in T2D mice and rats [6, 7] and ameliorate diabetic cardiomyopathy and nephropathy [8, 9]. Moreover, APSs can also ameliorate alcoholic fatty liver disease and hepatic endoplasmic reticulum (ER) stress [10, 11]. Therefore, possible improvement in NAFLD with APSs can be also expected. On the other hand, *Astragalus* saponins also possess potent antiglycation, antioxidant, and hepatoprotective activities beneficial for diabetes therapy [12, 13].

As mentioned, there is a close association between traditional indications and modern pharmacological evidence of the therapeutic value of this herb against diabetes. However, considering that the RA water extract (AM-W) is the traditional way of administration, two questions that remain to be answered are to determine the major bioactive principle in the RA decoction and whether the beneficial effects observed with purified APSs or saponins against T2D and NAFLD can be directly applied to the RA decoction.

Therefore, by employing T2D rats, the current investigation used the chemically defined RA water extract to evaluate possible impacts on insulin resistance, beta-cell function, and associated NAFLD in rats with T2D.

## 2. Subjects and Methods

### 2.1. Materials

Streptozotocin (STZ) and other chemicals were purchased from Sigma (St. Louis, MO, USA). Insulin was purchased from Novo Nordisk (Princeton, NJ, USA). The TRI reagent was purchased from Invitrogen (Taipei, Taiwan). Acetonitrile and methanol (LC grade) were purchased from Merck (Darmstadt, Germany). H4IIE cells were purchased from the Bioresource Collection and Research Center (BCRC, Taichung, Taiwan). BRIN-BD11 cells were obtained from Prof. Peter R. Flatt (University of Ulster, Coleraine, UK).

### 2.2. Plant Material and Preparation of RA Extracts

Dried roots of *Astragali membranaceus *were purchased as plant material from China and authenticated by Prof. Kuo. A voucher specimen was deposited at the National Research Institute of Chinese Medicine (Taipei, Taiwan). RA (600 g) was cut and ground into pieces of about 0.5 cm, then distilled and refluxed with 5 L of distilled water for 8 hours (h). The water extract (approximately 2.8 L) was filtered through 6 layers of cheesecloth and then put into a freeze-dryer to yield dried powder (AM-W; 198 g). In contrast, the ethanol extract was prepared from 100 g of RA with 0.5 L of 95% ethanol cooked at 50°C for 3 h three times. The final concentrated crude extract (AM-E) yielded 15.2 g. To characterize the polysaccharides, AM-W (7 g/dL) was submitted to precipitation with an equal volume of ethanol. Two fractions were obtained, and the upper layer was collected and concentrated as the first fraction, AM-W-F1. The precipitate was collected by centrifugation and became AM-W-F2 (2 g of precipitate) after washing with ethanol and acetone prior to drying in a vacuum oven.

### 2.3. Chemical Analysis by High-Performance Liquid Chromatography (HPLC) and Nuclear Magnetic Resonance (NMR)

By employing reference compounds including astragaloside I and II, isoastragaloside I, and sucrose, the AM-W and AM-E were analyzed using the following apparatus and conditions: the HPLC profile was obtained from a Shimadzu 10AVP series system equipped with two pumps (LC-10ADVP, Shimadzu, Kyoto, Japan), a column oven (CTO-10ASVP, Shimadzu), an evaporative light scattering detector (ELSD) (ELSD-LT II, Shimadzu), a two-channel vacuum degasser (model 2003, Biotech AB, Onsala, Sweden), an SIL-10AVP automatic injector, an SCL-10AVP system controller, and a class VP workstation for data analysis.

The procedure employed a separation column (Cosmosil 5C18-AR-II, 5 *μ*m, 4.6 × 250 mm I.D., Nacalai Tesque, Kyoto, Japan) eluted at a rate of 0.8 mL/min below 25°C. The mobile phase consisted of water (A) and acetonitrile (B) using a gradient program of 0% ~ 1% (B) at 0 ~ 10 min, 25% ~ 32% (B) at 10 ~ 25 min, 32% ~ 50% (B) at 25 ~ 50 min, and 50% ~ 100% (B) at 50 ~ 60 min. The temperature of the heated drift tube was 50°C, and the N_2_ gas flow-rate was 1.5 L/min for the ELSD detector. Equal amounts of sample solutions were filtered through 0.45-*μ*m filters (Millipore, Bedford, MA, USA) prior to the injection with a volume of 20 *μ*L.

For the polysaccharide and sucrose analyses, the NMR technique was employed by measuring ^1^H NMR and ^13^C NMR spectra of the test samples.

### 2.4. Generation of Insulin-Dependent Diabetes Mellitus (IDDM) and T2D Rats for AM-W Administration

Sprague-Dawley (SD) rats were purchased from the Animal Center of National Yang-Ming University, Taipei, Taiwan. This research followed the *Guide for the Care and Use of Laboratory Animals* (NIH publication, 85–23, revised 1996) and was approved by the Animal Research Committee at the NRICM. Adult male SD rats weighing 250 ~ 350 g were housed under controlled conditions (i.e., a 23°C room temperature, controlled humidity, and a light/dark cycle of 12/12 h). Induction of IDDM followed a previous description [14]. Briefly, SD rats were given two shots of STZ (100 and 50 mg/kg body weight) by an intraperitoneal (i.p.) injection on days 0 and 2, respectively. After 1 week, rats with a fasting blood glucose of >126 mg/dL were considered diabetic according to the definition of diabetes by the World Health Organization. Starved diabetic rats then received the vehicle, AM-W (500 and 1000 mg/kg), or insulin (2 U/kg). Blood sugar was monitored every 2 h for up to 10 h posttreatment.

The preparation of T2D rats was based on Sirnivasan et al.'s method with some modifications [15]. Briefly, SD rats were fed high-fat chow (high-fat diet) which was standard chow supplemented with extra lard (20%, w/w), cholesterol (1%, w/w), and cholic acid (0.1%, w/w). After 2 weeks of dietary manipulation, the high-fat-diet rats were i.p. injected with STZ (50 mg/kg). After 4 weeks, the rats were considered diabetic if the fasting blood sugar was >126 mg/dL.

Fourteen diabetic rats were randomly divided into two groups and received either prepared AM-W or saline (as the vehicle control). Daily i.p. administration was carried out with 500 mg/kg or an equivalent volume of saline for 2 months. Nine nondiabetic control rats fed the standard diet also received daily saline administration.

### 2.5. Intravenous Glucose Tolerance Test (IVGTT)

After overnight starving, glucose (1 g/kg) was injected via the tail vein, and blood sugar was measured at 0, 15, 30, 60, and 120 min throughout the test with commercial glucometers (Bioptik Technology, Taipei, Taiwan). The area under the curve (AUC) was calculated from the graph of blood glucose (mg/dL) versus time (min).

### 2.6. Serum Biochemical Analysis

Blood samples were collected from the tail vein of animals anesthetized with pentobarbital (30 mg/kg, i.p.). Serum triglyceride and total cholesterol were measured with a FUJI DRI-CHEM 3000 analyzer (Fuji Photo Film, Tokyo, Japan). Serum insulin was measured with an enzyme-linked immunosorbent assay (ELISA) kit (Millipore, Bedford, MA, USA). Both the homeostasis model assessment for insulin resistance (HOMA-IR = fasting blood glucose [mM] × fasting insulin [*μ*U/mL]/22.5) and HOMA for beta-cell function (HOMA-B = 20 × fasting insulin [*μ*U/mL]/Fasting blood glucose [mM] − 3.5) were calculated.

### 2.7. Pathological Examinations

At the end of the experiment, anesthetized animals were sacrificed in order to harvest the liver. A portion of liver was fixed with 4% (w/v) paraformaldehyde for a later paraffin or frozen-tissue section. For the qualitative pathological histology, hematoxylin and eosin (H&E) staining was used to illustrate the overall morphology. Oil red O (ORO) staining was used to identify the neutral lipid and fatty acid contents. A red color indicated intracellular triglycerides. Periodic acid-Schiff (PAS) staining was performed to determine the glycogen content. A purple color indicated cellular glycogen.

### 2.8. Western Blotting

The procedure followed a previous methodology [16]. In brief, tissues were immersed in ice-cold phosphate-buffered saline (PBS) prior to homogenization with an ice-cold lysis buffer. Tissue debris was removed by centrifugation. Equal amounts of protein (40 *μ*g) were subjected to separation on sodium dodecyl sulfate (SDS) 10% polyacrylamide gels. Following transfer to nitrocellulose membranes, blots were blocked with 5% (w/v) nonfat milk in Tris-buffered saline containing 0.1% (v/v) Tween 20 (TBST) for 1 h and incubated with primary antibodies at 4°C overnight prior to incubation for 1 h at room temperature with the secondary antibody. Finally, results were visualized after development of the film with the aid of an enhanced chemiluminescence (ECL) kit (Amersham Biosciences, Uppsala, Sweden). The intensity of the blots was quantified by AlphaEaseFC (Alpha Innotech, San Leandro, CA, USA).

### 2.9. Statistical Analysis

The effects of the three experimental manipulations on various parameters were evaluated by one-way analysis of variance (ANOVA) followed by the Tukey-Kramer test. Results are expressed as the mean ± S.E.M. Differences were considered significant at *P* < .05.

## 3. Results

### 3.1. Polysaccharides and Sucrose Are Two Major Constituents of the RA Decoction (AM-W)

**Figure 1:**
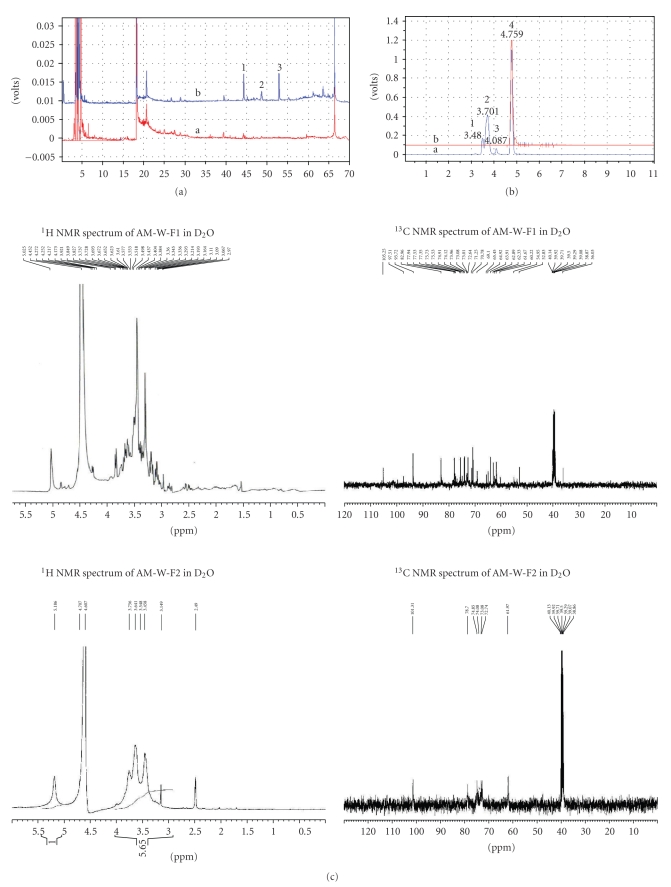

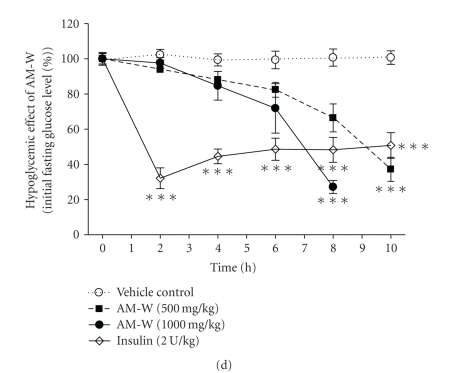
Polysaccharide and sucrose are only present in the water extract of Radix Astragali (RA) (AM-W). (a) LC-ELSD chromatograms of the AM-W (a) and the ethanolic extract (AM-E) (b) with a 70-min retention time. Peak identification: 1, astragaloside II; 2, isoastragaloside I; 3, astragaloside I. (b) LC-ELSD chromatograms of the AM-W (a) and sucrose (b) with a 10-min retention time. Peak identification: 1, 2, and 3, polysaccharides; peak 4, sucrose. (c) The representative ^1^H NMR and ^13^C NMR spectra of AM-W-F1 and AM-W-F2 are illustrated. (d) Confirmation of the hypoglycemic bioactivity of the AM-W. Insulin-dependent diabetic rats were generated with an initial average glucose level at 407.2 ± 17.8 mg/dL (*n* = 22). The hypoglycemic effect was evaluated by determining the percentage of the initial fasting glucose level in each group. Differences at the same time point were determined by ANOVA followed by the Tukey-Kramer test. *P < .05 was considered significant when compared to the control (vehicle) insulin-dependent diabetes mellitus rats.

From results of the ELSD detector, Figure 1(a) compares differences in chemical constituents of the water (AM-W) and ethanol extracts (AM-E). The chromatogram of AM-W indicated that most AM-W constituents were distributed in the highly polar (retention times (*t*_*R*_) of 0 ~ 15 min) and medium highly polar (*t*_*R*_ of 15 ~ 30 min, the flavonoid glycosides) zones, which differed from the AM-E chromatogram showing prominent peaks (*t*_*R*_ of 40 ~ 55 min) and components in the low polar zone (*t*_*R*_ of 45 ~ 67 min). Compared to authentic samples, this study further, respectively, identified peaks 1 ~ 3 from AM-E chromatogram as astragaloside II, isoastragaloside I, and astragaloside I. These results indicated that the AM-E is mainly composed of characteristic saponins and flavonoids.

In contrast, the AM-W possessed major components, including water-soluble oligosaccharides and polysaccharides which were further confirmed using HPLC and NMR. In Figure 1(b), peak 4 was determined to be sucrose (peak 4, *t*_*R*_ 4.759 min) with a relative amount of 53.78%, compared to the sucrose standard. In contrast, peaks 1 ~ 3 were polysaccharides with relative amounts as follows: peak 1 (*t*_*R*_ 3.480 min, 8.09%), peak 2 (*t*_*R*_ 3.701 min, 34.92%), and peak 3 (*t*_*R*_ 4.087 min, 3.21%).

In Figure 1(c), the ^1^H NMR and ^13^C NMR spectra of AM-W-F1 were very similar to the characteristic signals of the sucrose standard. In contrast, the ^1^H and ^13^C NMR spectra of AM-W-F2 contained distinctive broad signals which indicated the presence of polysaccharides [17] which belong to the structure of 1,4-*α*-D-glucan [*δ*C 101.3 (C-1), 78.7(C-4), 74.9 (C-3), 73.1 (C-2), 72.7 (C-5), 62.0 (C-6)].

In Figure 1(d), while IDDM rats injected with saline showed no effects on the fasting blood glucose level, administration of AM-W to IDDM rats resulted in a dose-dependent blood sugar reduction within 10 h of treatment. In contrast, an insulin injection led to an acute antihyperglycemic effect within 2 h of treatment which lasted for 10 h.

### 3.2. Therapeutic Efficacy of AM-W Administration against T2D

**Figure 2:**
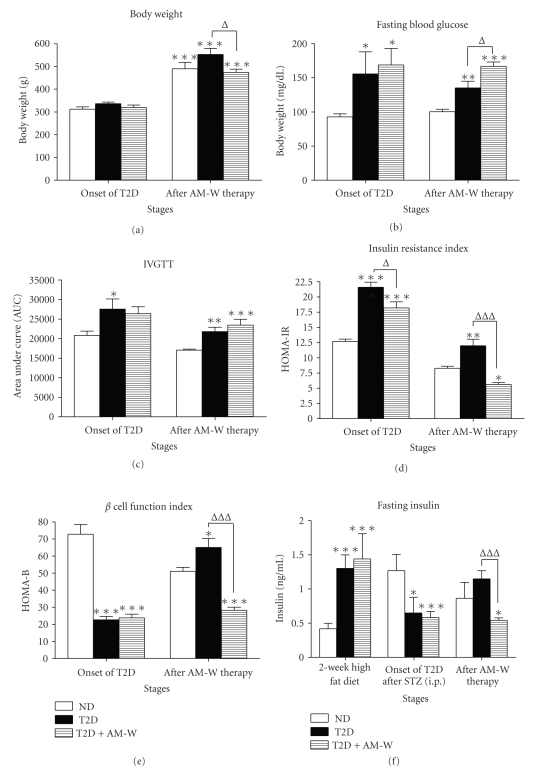
Therapeutic efficacy of the water extract of Radix Astragali (AM-W) for type 2 diabetes (T2D). Once induced T2D rats were identified, the following biochemical parameters were evaluated before and after 2 months of AM-W treatment. (a) Body weight (g); (b) fasting blood sugar (mg/dL); (c) intravenous glucose tolerance test (IVGTT) for which results were calculated as the area under the curve (AUC); (d) the insulin resistance index for which results are presented as HOMA-IR; (e) beta-cell function index for which results are presented as HOMA-IR; (f) fasting serum insulin (ng/mL). Differences were determined by ANOVA followed by the Tukey-Kramer test. For body weight, *P < .05 was considered significant when compared to the body weight before AM-W treatment. For the remaining parameters, *P < .05 compared to the nondiabetic group (ND) and ^Δ^ *P* < .05 for the comparison between T2D and T2D + AM-W (500 mg/kg) rats.

According to the Chinese herbal drug safety information published by the Committee on Chinese Medicine and Pharmacy (CCMP), Department of Health (DOH), Taiwan, rats receiving an i.p. injection of 500 mg/kg of the RA decoction for 1 month should experience nonpathological weight loss due to a reduction in food intake [18]. As a result, a 2-month administration dosage (AM-W at 500 mg/kg; equivalent to 230 mg APS/kg) for T2D rats was executed. A detailed evaluation of the therapeutic efficacy is illustrated in Figure 2. In Figure 2(a), the body weight of all three groups significantly increased in the 2-month period. However, it was noted that rats in the T2D vehicle group grew faster than those in the nondiabetic group (ND) vehicle group. Consistently with the CCMP report, administration of AM-W for T2D significantly reduced the body weight gain compared to untreated T2D rats, and they reached a level similar to that of the ND group.

In terms of glycemic control, to our surprise, fasting high blood glucose level in T2D rats did not decrease but was elevated in the T2D + AM-W group (^Δ^ *P* < .05) (Figure 2(b)). In addition, IVGTT observed in T2D rats did not improve with AM-W administration (Figure 2(c)). However, the increase in HOMA-IR in T2D rats was significantly alleviated by AM-W (^∆∆∆^ *P* < .01; Figure 2(d)). In contrast, HOMA-B in T2D rats was actually reduced by AM-W treatment (^∆∆∆^ *P* < .01; Figure 2(e)). By examining changes in the fasting insulin level at various stages, it was clearly illustrated that hyperinsulinemia was induced by the high-fat diet, and impaired insulin secretion was achieved with a low-dose STZ injection (Figure 2(f)). However, although both insulin secretion and beta-cell function gradually recovered during the 2-month period in untreated T2D rats, AM-W administration appeared to lead to the abolishment of such a recovery process in T2D rats (Figures 2(e) and 2(f)).

**Figure 3:**
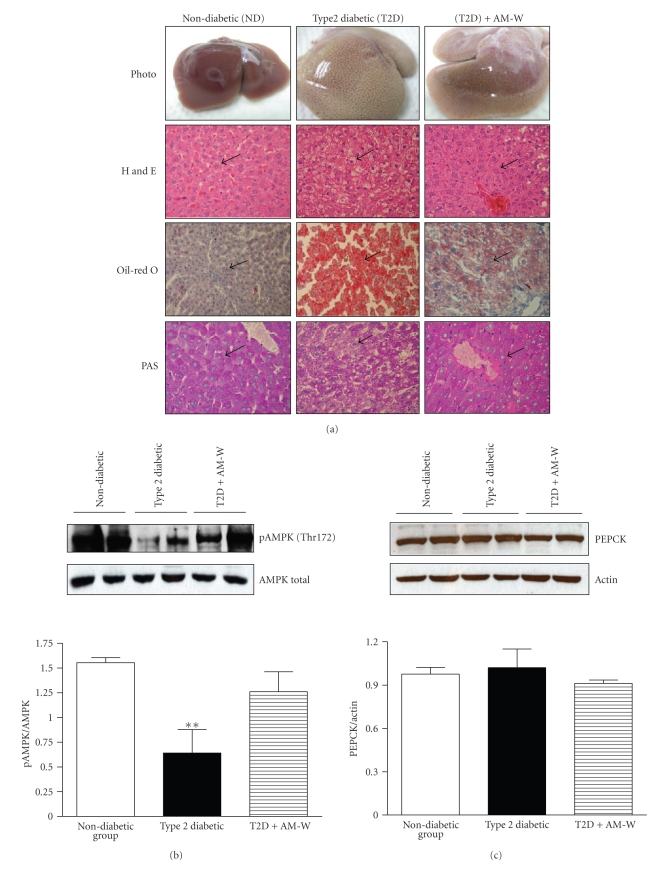

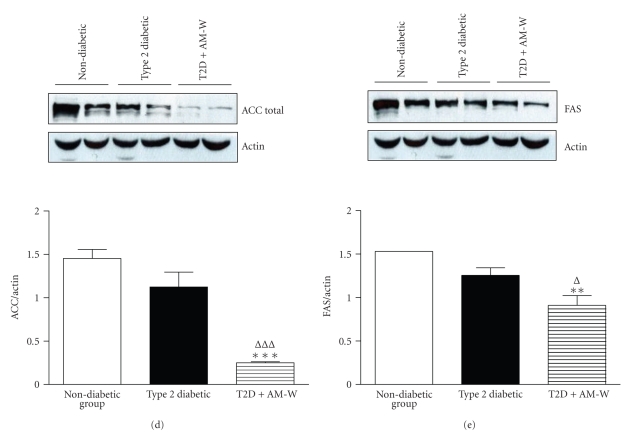
Liver histopathology and hepatic protein level analysis after the water extract of Radix Astragali (AM-W) administration. (a) At the end of AM-W administration, representative liver histopathology was examined and illustrated. Representative pictures from photography, H&E staining, oil-red O staining, periodic acid-Schiff staining, and Masson's trichrome of control (ND), type 2 diabetes (T2D), and T2D + AM-W groups are shown. In addition, hepatic proteins from each group were extracted to measure the expressions of (b) AMP kinase (AMPK) phosphorylated at Thr172 (pAMPK), (c) phosphoenolpyruvate carboxykinase (PEPCK), (d) acetyl CoA carboxylase (ACC), and (e) fatty acid synthase (FAS). Each blot was quantified, converted to the relative ratio of actin, and presented as the mean ± SEM (*n* ≥ 3 in each group). Differences were determined by ANOVA followed by the Tukey-Kramer test. *P < .05 was considered significant when compared to ND rats. ^Δ^ *P* < .05 was considered significant when T2D and T2D + AM-W rats were compared.

As shown in Figure 3(a), hepatocytes in rats fed with standard diet presented polyhedral morphology with eosinophilic cytoplasm and a usually central nucleus. In contrast, hepatocytes in T2D rats possessed diffuse microvesicular steatosis characteristics and showed loss of cytoplasmic eosin. In addition, nucleus was forced to diverge from central location. AM-W administration attenuated such pathological histology observed in T2D. Consistently, substantial amount of intracellular lipid was stained by Oil red O staining method in the liver section of untreated T2D while no obvious Oil red O stain could be observed in the liver section of nondiabetic control. AM-W administration significantly reduced such intracellular lipid accumulation in T2D rats. Finally glycogen content (as judged by PAS staining) was also depleted in untreated T2D rats compared to the nondiabetic controls and attenuated in T2D rats by AM-W administration.

 By examining hepatic protein expression, Figure 3(b) shows that AMPK activity was inhibited in T2D rats as judged by a reduction in pAMPK expression compared to ND rats. AM-W administration was associated with pAMPK expression at a level similar to that in ND rats. In addition, while phosphoenolpyruvate carboxykinase (PEPCK) protein expression was unchanged (Figure 3(c)), both acetyl-CoA carboxylase (ACC) and fatty acid synthase (FAS) protein expressions were reduced in T2D + AM-W rats (Figures 3(d) and 3(e)).

### 3.3. Counteracting Effects of the Combination of Sucrose and APS on the Viability and Glucose Response of BRIN-BD11 Cells

**Figure 4:**
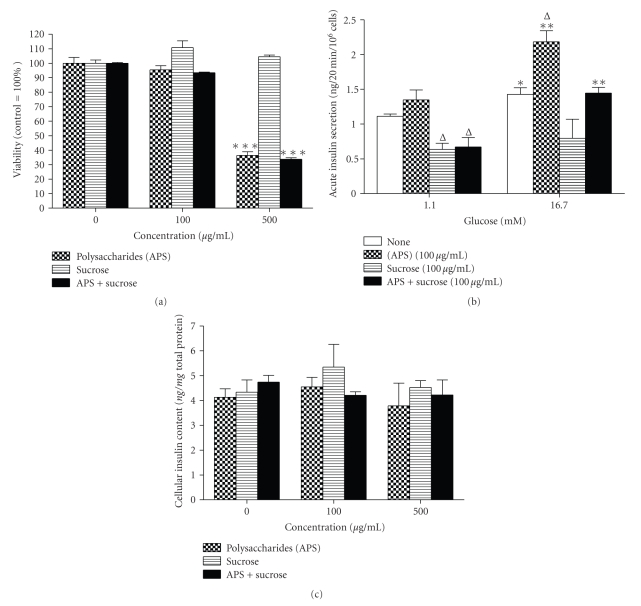
The effects of *Astragalus* polysaccharides (APS) and sucrose alone or in combination on the viability, insulin secretion, and cellular insulin content of BRIN-BD11 cells at 48 h posttreatment. After treatment for 48 h, (a) the cell viability, (b) glucose-stimulated insulin secretion, and (c) cellular insulin content of BRIN-BD11 cells were measured. Data are presented as the mean ± SEM (*n* ≥ 4 in each group). *P < .05 was considered significant compared to the viability of untreated control or respective basal insulin secretion (1.1 mM glucose). ^Δ^ *P* < .05 was considered significant compared to the insulin secretion of the untreated control.

In Figure 4(a), a significant reduction of cell viability (**P < .01) was observed in BRIN-BD11 cells cultured with either APS (500 *μ*g/mL) alone or in combination with sucrose (500 *μ*g/mL) for 48 h. In Figure 4(b), the presence of APS (100 *μ*g/mL) alone enhanced insulin secretion in response to high glucose (16.7 mM) compared to the normal culture control. However, the presence of sucrose (100 *μ*g/mL) led to a reduction in basal insulin secretion and the abolishment of glucose responsiveness. In addition, the beneficial effect of APS on glucose responsiveness was also compromised in the presence of sucrose (Figure 4(b)). Finally, the above culture conditions did not influence the cellular insulin content of BRIN-BD11 cells (Figure 4(c)).

## 4. Discussion

The RA decoction is traditionally prepared by water extraction. Different from ethanol extraction which contains mainly nonpolysaccharides, APSs appear to be the major bioactive constituent, at approximately 30% ~ 47% (w/w), in the RA decoction (AM-W). On the other hand, a significant amount of sucrose, approximately 50% (w/w), also exists in the AM-W. By employing IDDM rats, the dose-dependent hypoglycemic activity of the AM-W was confirmed in the current investigation. Such a finding is consistent with another report [9] and suggested that different from insulin's action, the immediate hypoglycemic effect of the AM-W was mild but unaffected by the presence of sucrose.

In terms of administration routes for *Astragalus* decoction or its bioconstituents, compared with oral administration, injection method appeared to provide similar bioactivities. For example, consistent with the traditional indications that RA consumption could stimulate the immune system and improve cardiovascular health, clinical *Astragalus* injection promoted the recovery of hemopoietic function in patients with chronic aplastic anemia [19]. Incidence of cardiac events was also lower in congestive heart failure (CHF) patients being given *Astragalus* injection [20]. Moreover, reduced amounts of RA decoction or RA constituents via injection could be employed to achieve similar bioactivity provided by oral administration [21, 22]. As a result, i.p. injection was decided to be employed for current experimental model of T2D. After administering the AM-W for 2 months, the current results demonstrated that various pharmacological effects of the AM-W were consistent with previous works, including the insulin-sensitizing effect, stabilization of serum cholesterol (data not shown), and attenuation of hepatic triglyceride accumulation and glycogen loss in T2D rats [6, 10, 18]. Consistent with Zou et al.'s observations, the level of activated AMPK decreased in skeletal muscle of diabetic rats induced by a high-fat diet plus STZ [23]. In the present study, results also showed that the level of activated AMPK in the liver was less in T2D rats, whereas AM-W administration was associated with greater activated AMPK levels. Therefore, our data agree with previous findings that APS administration results in both glycogen resynthesis and improved AMPK activities in T2D rats [23]. In addition, combining the observations that AM-W administration repressed both ACC and FAS expressions [24], the current results suggest that attenuation of fatty liver by the AM-W might be a result of suppression at proximal steps of hepatic lipogenesis and restoration of normal fatty acid oxidation activity [25].

However, to our surprise, the fasting blood glucose level of T2D + AM-W rats had not improved after 2 months of treatment. Moreover, despite the beneficial effects of APS on both the beta-cell mass and function in type 1 diabetes reported by others [5, 26], the current results indicated that beta-cell function deteriorated after AM-W administration. We speculated that the excessive sucrose content in the AM-W might be the key reason for the reduction in beta-cell function after AM-W treatment, because various studies demonstrated the deleterious effects of sucrose and fructose consumption on beta-cell mass and functions [27–29]. By employing glucose-responsive BRIN-BD11 cells, the impact of the presence of sucrose on glucose-stimulated insulin secretion was evident. Moreover, the beneficial effect on BRIN-BD11 cells provided by APSs was also compromised by the presence of an equal amount of sucrose. Therefore, the possibility that administration of sucrose-rich AM-W led to a worsening of beta-cell function in T2D rats exists. However, a reduction in cell viability was also observed in the presence of a high concentration of APSs. Although the APS content in the current investigation was much lower than that of any previous report, we still could not rule out the potential influence of APSs in the AM-W on the pancreatic beta-cell mass in T2D rats.

 RA (*Huang Qi*) is a very important herb in TCM applications and is frequently used as a dietary supplement. A statistical analysis of TCM prescription frequency for diabetes therapy from the database of the Taiwan National Health Insurance (2002-2003) also revealed that RA is the second highest single-drug to be prescribed for caring for DM outpatients at TCM clinics in Taiwan [30]. In China, 6 of 7 herbal drug products, containing RA as one of the ingredients, were approved by the Chinese health regulatory agency for commercial purposes [31]. Our investigation indicated that the RA decoction possesses several antidiabetic characteristics similar to those of purified APSs. As a result, it should be safe to say that consumption of the RA decoction could benefit type 2 diabetics in some way. However, the current investigation also pointed out the potential deleterious effect of the AM-W on beta-cell function due to the coexistence of sucrose. To our knowledge, this is the first report investigating the potential impact of the two major constituents in the RA decoction on beta-cell functions. Nevertheless, it should also be realized that the consistent use of a single herb (the RA decoction) for diabetes intervention is a very rare situation. Whether such a high sucrose content exists in commercialized TCM products remains unknown. Yet, according to the current results, it would be logical to expect that the potency and consistency of RA-containing TCM formulae could be further enhanced by removing sucrose from those decoctions.

In conclusion, the RA decoction contains beneficial APSs for T2D management and can be also developed as an anti-NAFLD agent. To achieve consistent efficacy, employing purified *Astragalus* polysaccharides or a sucrose-depleted RA*-*included decoction might be a good option to maximize the merits of such a valuable TCM herb.
